# Sensing Through Tissues Using Diffuse Optical Imaging and Genetic Programming

**DOI:** 10.3390/s26010318

**Published:** 2026-01-03

**Authors:** Ganesh M. Balasubramaniam, Ami Hauptman, Shlomi Arnon

**Affiliations:** Department of Electrical and Computer Engineering, Ben-Gurion University of the Negev, Be’er Sheva 8441405, Israel; ganeshb@post.bgu.ac.il (G.M.B.); amih@afeka.ac.il (A.H.)

**Keywords:** sensing, diffuse optical imaging, genetic programming, image reconstruction, diffuse media, inverse problems, machine learning

## Abstract

Diffuse optical imaging (DOI) uses scattered light to non-invasively sense and image highly diffuse media, including biological tissues such as the breast and brain. Despite its clinical potential, widespread adoption remains limited because physical constraints, limited available datasets, and conventional reconstruction algorithms struggle with the strongly nonlinear, ill-posed inverse problem posed by multiple photon scattering. We introduce Diffuse optical Imaging using Genetic Programming (DI-GP), a physics-guided and fully interpretable genetic programming framework for DOI. Grounded in the diffusion equation, DI-GP evolves closed-form symbolic mappings that enable fast and accurate 2-D reconstructions in strongly scattering media. Unlike deep neural networks, Genetic Programming (GP) naturally produces symbolic expressions, explicit rules, and transparent computational pipelines—an increasingly important capability as regulatory and high-stakes domains (e.g., FDA/EMA, medical imaging regulation) demand explainable and auditable AI systems, and where training data are often scarce. DI-GP delivers substantially faster inference and improved qualitative and quantitative reconstruction performance compared to analytical baselines. We validate the approach in both simulations and tabletop experiments, recovering targets without prior knowledge of shape or location at depths exceeding ~25 transport mean-free paths. Additional experiments demonstrate centimeter-scale imaging in tissue-like media, highlighting the promise of DI-GP for non-invasive deep-tissue imaging and its potential as a foundation for practical DOI systems.

## 1. Introduction

Diffuse optical imaging (DOI) is a non-invasive computational image retrieval technique employing near-infrared (NIR) light to explore thick scattering media like biological tissues or fog [1,2,3,4,5]. NIR light can penetrate several centimeters into soft tissues like the breast and brain [6]. Because of this capability, DOI has diverse medical applications, including tumor detection [7,8], brain studies [9,10,11,12], and chemotherapy treatment monitoring [13,14,15]. Its safety and ability to provide detailed tissue information make DOI a promising tool for diagnosing diseases and guiding treatment strategies [16,17].

Numerous specialized mathematical and computational algorithms have been developed for reconstructing anomalies within diffuse media with diverse geometrical and physical properties over recent decades [17,18,19,20]. However, image reconstruction in this domain poses significant challenges, primarily due to the inherent complexity of the inverse problem [21]. The inverse problem is nonlinear, ill-posed, and ill-conditioned [17,22]. Furthermore, while camera measurements of light transmission through diffuse media offer notable imaging contrast, the reconstructed images often exhibit subpar spatial resolution [23,24]. Additionally, many classical methods rely on assumptions of complete measurements or strong linearization, usually based on precisely known boundary conditions, a scenario seldom achievable in practical settings. This limitation arises from the nature of light propagation within diffuse media, where the light takes a convoluted and unpredictable path influenced by multiple scattering events [25].

In recent years, addressing this challenge has spurred active research into employing various deep neural network (DNN) architectures for image reconstruction in diffuse optical imaging [13,26,27,28,29]. These algorithms offer several advantages for image reconstruction in DOI. They excel at learning complex mappings between input (camera measurement) and output (reconstructed anomalies) spaces, allowing for more accurate and robust reconstruction even in the presence of noise and uncertainties [30,31,32]. Additionally, deep neural networks can capture intricate patterns and relationships within the data, enabling enhanced spatial resolution and finer detail in the reconstructed images compared to traditional methods [16,26]. Moreover, deep learning approaches have the flexibility to adapt to different imaging scenarios and can be trained on diverse datasets, making them highly versatile for DOI applications across various tissue types and conditions [13].

However, despite their promise, deep learning methods for DOI image reconstruction have certain limitations. These include the need for large training data, which may be challenging to acquire, especially for rare or specialized imaging scenarios. Additionally, deep neural networks can be computationally intensive, requiring substantial resources for training and inference, which may limit their practical implementation in real-time imaging systems. Moreover, the “black-box” nature of several deep learning models can make it difficult to interpret the reasoning behind their decisions, raising concerns about the reliability and trustworthiness of the reconstructed images in clinical settings [16,17,27,33].

Some of the above limitations associated with learning-based approaches like DNNs can be mitigated through evolution-based methods such as Genetic Programming (GP) [34]. Genetic programming is a powerful computational technique inspired by biological evolution that automatically generates tree-like recursive computer programs to solve complex problems [35]. By mimicking the process of natural selection, GP evolves a population of candidate solutions over successive generations, repeatedly applying genetic operators such as mutation, crossover, and selection to facilitate evolutionary search [34,36]. This continual optimization process allows GP to effectively search large solution spaces and discover high-performing solutions. Modern science and medicine increasingly require transparent and interpretable AI models rather than opaque black-box systems. Genetic Programming (GP) naturally produces symbolic expressions, explicit rules, and fully interpretable computational pipelines, offering transparency by design. Unlike deep neural networks—which often depend on post hoc explanation techniques—GP provides built-in interpretability. This capability is especially relevant today, as regulatory and high-stakes domains such as the FDA, EMA, and medical imaging now mandate explainable and auditable AI systems. GP aligns directly with these requirements, making it a strong and compliant alternative to black-box approaches. Moreover, many real-world “AI for science” problems operate in low-data, noisy, or incomplete-data environments, where traditional deep learning struggles. GP excels under these conditions, delivering robust performance with limited samples while enabling flexible, globally optimized, and physically grounded model discovery. Together, these strengths—interpretability, flexibility, global optimization, symbolic discovery, and strong low-data performance—explain why GP is increasingly relevant in modern scientific and medical AI. To summarize, GP presents several advantages. Firstly, because the programs evolved by GP may contain functions tailored to the specific problem domain, the process often demands less training data since it leverages existing domain knowledge rather than constructing models from scratch. This attribute translates into shorter training times, particularly suitable for applications requiring swift deployment [37]. Furthermore, GP trees are comprehensible to human programmers in certain conditions, offering a distinct advantage in interpretability compared to the often opaque neural networks. Such transparency is crucial, especially in fields like medical imaging, where comprehension and trust in the reconstruction process are paramount [38]. Additionally, GP’s adaptability in defining and refining the cost function enables customization to the specific requirements and constraints of the task, unlike the more rigid loss functions typical in learning algorithms [39]. These characteristics allow GP to find utility and relevance in various applications and industries [37,38,40].

GP presents a unique approach to image reconstruction, focusing on evolving executable structures rather than specific parameter values [37]. GP’s adaptability in representing and evolving executable code enables it to discern complex relationships within image datasets, thereby enhancing reconstruction accuracy. Moreover, GP’s interpretability and transparent modeling approach render it suitable for applications necessitating explainable AI, such as forensic imaging and industrial quality control [41,42,43]. These recent advancements highlight GP’s growing significance in image reconstruction research and its potential for addressing real-world challenges across various domains [37,40,44,45].

We introduce Diffuse optical Imaging using Genetic Programming (DI-GP), a novel GP algorithm tailored to efficiently and accurately reconstruct 2D objects in strongly scattering media. Unlike recent machine learning (ML) approaches, which often require extensive computational resources, large datasets, and lengthy training times to achieve desired outcomes, DI-GP focuses on minimizing training duration, reducing dependence on extensive datasets, and effectively transferring domain knowledge to real-world experiments. DI-GP achieves this by directly integrating the principles of the diffusion equation into its evolutionary process, allowing for the development of optimized solutions with greater precision and efficiency [25].

In addition to the advantages over DNNs outlined earlier, the proposed approach also presents several notable benefits compared to traditional analytical methods. These include expedited inference times and superior performance across multiple qualitative and quantitative metrics, such as a mean squared error of 0.0240 ± 0.0121, a structural similarity index of 0.8108 ± 0.0498, and a Pearson correlation coefficient of 0.7919 ± 0.0866. Furthermore, our algorithm can retrieve objects at imaging depths corresponding to several centimeters of human tissue [46]. Overall, our findings underscore the potential of DI-GP as a valuable tool for rapid and accurate reconstruction in DOI applications as a proof of concept.

The following sections detail the image reconstruction algorithm, elaborate on the experimental methodology, and present the results obtained from our study.

## 2. Theory, Methods and Experiments

This section details the methodology of computational imaging in the diffuse regime. We start by discussing the forward and inverse problems associated with this imaging technique. Following this, we explore the methods for simulating light transport and collecting training data for algorithm development. Then, we introduce DI-GP, a novel approach that utilizes Genetic Programming for computational diffuse optical imaging. Finally, we provide a detailed overview of the experimental setup and procedures employed to validate the effectiveness of our proposed methodology.

### 2.1. Forward and Inverse Problems in Diffuse Optical Imaging

The forward problem in computational diffuse optical imaging involves predicting light distribution within the diffuse media based on known optical properties and boundary conditions. We conducted experiments using a melamine sponge with a thickness of 3 cm, where the transport mean free path (τ) is more than 25 times smaller (at λ = 639 nm) than the total thickness of the material. This configuration places the light in the strongly diffuse regime, enabling us to utilize a diffusion approximation to model the propagation of photons within the diffuse medium [24,25]. The diffusion equation can be expressed as:

(1) $$\mu_{a}\Phi \left(\vec{r}\right)-\nabla \cdot \left[D\nabla \Phi \left(\vec{r}\right)\right]=S\left(\vec{r}\right)$$

Here, $\Phi \left(\vec{r}\right)$ represents the photon flux at a position r, $\mu_{a}$ is the absorption coefficient, $S\left(\vec{r}\right)$ is the source power density, and D is the diffuse coefficient given by ${D=\left(3\left(\mu_{a}+\mu_{s}^{\prime }\right)\right)}^{-1}$. Here, $\mu_{s}^{\prime }$ is the reduced scattering coefficient and is given by $\mu_{s}\left(1-g\right)$, where $\mu_{s}$ denotes the scattering coefficient and *g* denotes the anisotropy factor, given by the mean cosine of the scattering angle. The reduced scattering coefficient captures the effective rate of directional randomization in anisotropic scattering media.

When photon propagation is modeled using Equation (1), it follows the steepest descent of the scalar gradient weighted by the diffusivity (dominated by scattering $\mu_{s}$ in strongly diffuse media), accompanied by an additional loss effect due to photon absorption. For highly localized sources, Equation (1) has an analytical solution given by [25]:

(2) $$\Phi_{m}\left(\vec{r}\right)=\varphi \frac{e^{-\left\{\left(\sqrt{\frac{\mu_{a}}{D}}\right)r\right\}}}{4\pi Drc}$$

Here, $\Phi_{m}$ is the photon flux measurement at distance r, φ is the source amplitude term, and c is the speed of light in the medium.

Solving the forward problem is crucial for comprehending light interactions within diffuse media and serves as the foundation for image reconstruction in diffuse optical imaging [17,19,47]. In computational diffuse optical imaging, the inverse problem revolves around deducing the characteristics of hidden structures within a medium based on measurements obtained from the camera. This problem is particularly pertinent in scenarios where the goal is to reconstruct the shape, contrast, or other relevant parameters of objects embedded within biological tissue or other scattering media [23,48].

In scenarios where anomalies are present within an otherwise homogeneous background, the anomaly can be reconstructed by inverting a linearized forward model given by:

(3) M=J·χ

Here, M is the measurement obtained at the camera, χ is the optical perturbation, and J is the Jacobian matrix which is given by $J=\partial \Phi \left(\vec{r}\right)/\partial \mu_{a}$, and can be obtained from Equation (2) [25].

Initially, we compute the forward model using Equation (2) to simulate how light travels from the input plane to the object plane. Then, we use simple estimates to mask the propagation from the object plane in the diffuse medium to the measurement matrix. Next, we compare this numerical solution to the actual measurement by checking a cost function. This function helps adjust the shape of the estimated object, refining it through an iterative process of solving Equation (3) with the adjusted guess. Therefore, computational image reconstruction in diffuse optical imaging is typically framed as an optimization task aimed at minimizing the objective function given by:

(4) $${argmin}_{\chi }\left({\left\|J\cdot \chi -M\right\|}^{2}\right)+\delta$$

Here, δ represents the regularization term, frequently derived from specific assumptions regarding the statistical properties of optical images [16,29,49]. Similarly, in this article, we propose a GP algorithm that retrieves anomalies (χ) inside a strongly diffuse medium by optimizing a modified Equation (4) given by:

(5) $$\chi_{retrieved}=\left\{{argmin}_{\chi }\left(\frac{1}{2}{\left\|J{\cdot \chi }_{predction}-M\right\|}_{2}^{2}\right)+\delta \right\}\times T\left(M\right)$$

Here, $\chi_{retrieved}$ is the retrieved image containing the anomaly distribution, $\chi_{predction}$ is the predicted outcome for each individual of the GP algorithm and $T\left(M\right)$ is the thresholding mask, which works by minimizing Equation (5) only over pixels where the signal-to-noise ratio in the recorded data M exceeds a certain threshold [23]. The binary mask defined from a per-pixel signal-to-noise ratio map SNRx,y=Mx,yσn, where $\sigma_{n}$ denotes the standard deviation of background noise estimated from reference measurements acquired without the anomaly. Pixels satisfying $SNR\left(x,y\right)\geq {SNR}_{thr}$ are retained, whereas the remaining pixels are excluded from the minimization to prevent noise-dominated measurements from influencing the solution. In this work, ${SNR}_{thr}$ denotes the threshold SNR which is set at ${SNR}_{thr}=3$.

The details of the algorithm are presented in Section 2.3. However, before delving into the image reconstruction algorithm, we look at the light transport simulations and the dataset used to evolve the GP algorithm.

### 2.2. Light Transport Simulations and Training Data

Applying evolution-based methods for DOI reconstruction requires physical knowledge about the background sample and the embedded anomalies, contributing to the contrast observed in DOI measurements. This necessitates the availability of a dataset. Although GP algorithms require datasets that are several orders of magnitude smaller than DNNs, acquiring such datasets in the context of DOI remains challenging [13,37,40]. Therefore, simulating light propagation through digital phantoms offers a viable approach to generating datasets for algorithm training. Furthermore, light transport simulations offer another advantage: they allow for the assessment of experimental design considerations through computer simulations, helping to avoid the costly and wasteful construction of clinical prototype systems that may possess inherent design flaws [8,18].

**Figure 1:**
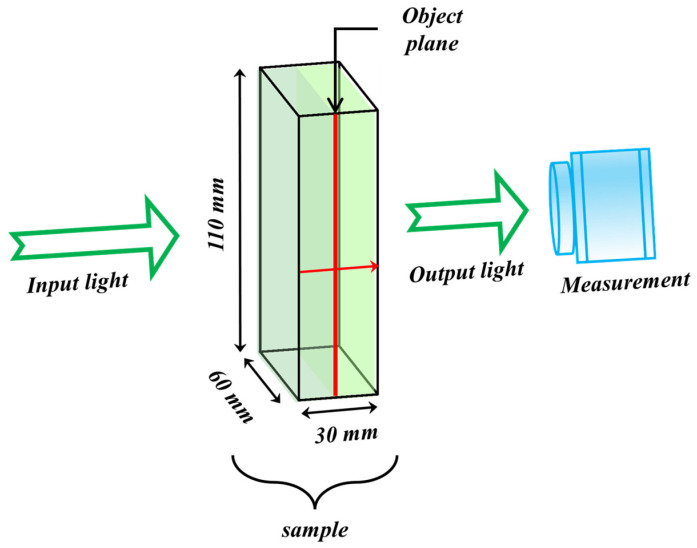
Schematic of the light transport simulations performed in this study. The red arrow within the volumetric mesh denotes the direction of photon propagation.

In this study, simulations are performed using a cuboid volume measuring 110 × 60 × 30 ${mm}^{3}$ (height × width × thickness), as depicted in Figure 1. The volumetric mesh is generated using a MATLAB-based meshing tool called iso2mesh [50]. The sample’s imaging thickness is 30 mm, with absorption and scattering coefficients of 0.0035 ${mm}^{-1}$ and 0.8090 ${mm}^{-1}$, respectively. These parameters were derived by fitting experimental measurements to the diffusion equation [23,51,52].

A continuous wave Gaussian light source operating at a wavelength of 639 nm and a spot size of radius 1.5 cm is employed to illuminate the sample, with flux measurements taken at the opposite boundary. Anomalies are introduced at the central depth of the sample (15 mm), comprising hand-drawn shapes placed within the field of view of the imaging system. These anomalies are characterized by absorption coefficients ranging from 0.5 ${mm}^{-1}$ to 0.72 ${mm}^{-1}$, representing the contrast between the anomalies and background samples in the experimental material.

The light transport simulations were executed using MCX [53,54]. This MATLAB-based software employs Monte Carlo algorithms to model light transport in diffuse media. The simulations were conducted on 30 distinct meshes, each featuring a unique hand-drawn anomaly shape. For each shape, both the shape itself and the corresponding measurement data were saved, resulting in a dataset comprising 30 measurement vectors as inputs and the corresponding anomaly distributions as ground truths. Additionally, the Jacobian at the imaging plane and the reference measurements were obtained by conducting simulations without the anomalies. A 2% Gaussian noise is added to the measurements to simulate any experimental noise [8,55]. The dataset is utilized to train the DI-GP, which evolves an optimal solution for retrieving hidden anomalies within diffuse media. The algorithm is described in the next section.

### 2.3. Training the DI-GP Algorithm: Diffuse Optical Imaging Using Genetic Programming

**Figure 2:**
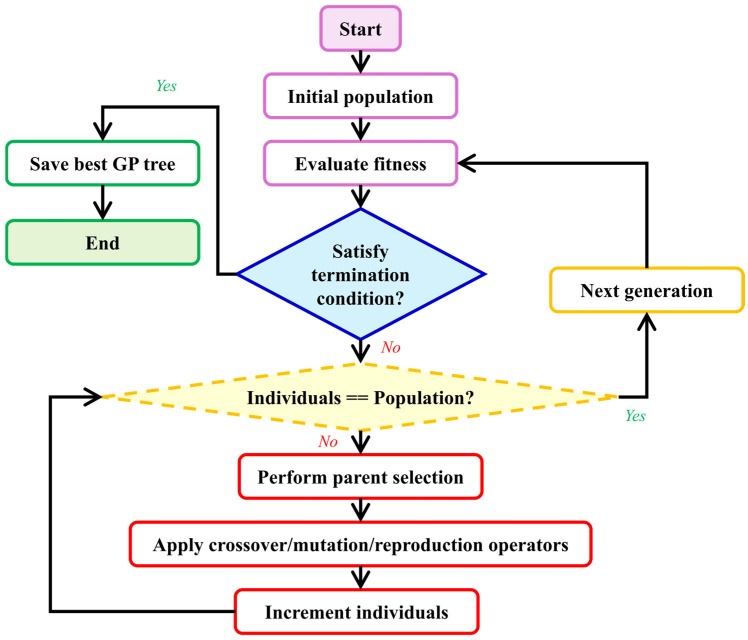
Schematic of a generic Genetic Programming algorithm.

Genetic Programming is a nature-inspired hyper-heuristic search algorithm that generates and evolves computer programs representing solutions to a given problem. GP begins with a set of basic building blocks, a high-level objective, and a method for evaluating solution quality. As illustrated in Figure 2, it automatically generates a group (or population) of random initial solutions (or individuals). Individuals are computer programs structured as trees. This structure allows for a hierarchical organization of operations and data, resembling branches and leaves, where each node represents a computational step or decision point. Individuals are evaluated using a predefined fitness function specifically tailored to the task at hand [35,56]. Better individuals are given more favorable fitness scores.

GP then constructs the next population of individuals (or the next generation) by using a method analogous to natural selection: better individuals (i.e., those with better fitness scores) are selected with a higher probability for the application of genetic operators, such as crossover, mutation, and gene duplication [35]. Through fitness-based selections and genetic operators applied over time, genetic algorithms iteratively optimize solutions during simulated evolution.

Each genetic operator yields one or more individuals, which are inserted into the population. In contrast, individuals who were not selected are discarded, adhering to the survival of the fittest principle. The process then reiterates until one of the termination conditions is met, typically after a fixed number of generations has passed. Thus, over successive generations, through fitness-based selections and genetic operators applied repeatedly over time, genetic algorithms refine and optimize solutions to the specified problem. Further details regarding the image reconstruction are provided in the following sub-sections.

#### 2.3.1. Image Reconstruction Using Genetic Programming and Structure of the GP Individuals

In this study, we utilize a Koza-style GP [34] within the DEAP Python environment [57]. In this framework, each GP individual in our algorithm embodies a function of the form $R^{N\times N}\to R$, where it processes the inputs represent a neighborhood of N×N (N = 5 in our case) pixels from the measurement image (M) and produces a single pixel at a corresponding location in the predicted image ($\chi_{predction}$). This is repeated for all pixels in M where the N×N environment is fully contained in the image, while pixels with inadequate environments, in the margins or corners, are skipped. Consequently, the image is reconstructed pixel by pixel, leveraging local pixel data of the measurements. The pseudocode in Table 1 represents the generation of a predicted image where *M* is the input image, *GP_individual* is the individual currently being evaluated, *N* is the width of the environment (the environment is N×N pixels).

GP individuals, serving as candidate solutions for reconstructing a pixel using a local NxN environment of pixels in M, embody functions of the form $R^{N\times N}\to R,$ as stated above. Each GP individual (or GP tree) resembles a recursive LISP expression, consisting of inner functions that accept arguments and terminals which serve as leaf nodes without any arguments [58]. Trees are initially constructed at random, adhering to specific depth constraints (see below). The leaf nodes are either input pixels, marked as $Pixel_{1}$, $Pixel_{2}$, …, $Pixel_{N\times N}$, or random constants in the range [0, 1]. Inner tree-nodes are functions with varying arities, with each being applied to the appropriate number of inputs, which can be outputs of other tree-nodes or values from terminals. All functions and terminals output numeric values. The final values GP individuals return are clipped to the range [0, 1]. Table 2 provides a comprehensive list of inner functions and terminals utilized by GP.

#### 2.3.2. Parameters for Evolution

The algorithm’s parameters were empirically defined within specific ranges to optimize performance. The population size, representing the number of individuals in each generation, varied between 150 and 250, which is relatively small but still provides enough diversity. Generation count, indicating the number of iterations or generations, ranged from 20 to 40 since evolution typically converged to good solutions relatively fast. The crossover probability was set at 0.6 and the mutation probability at 0.4, which is relatively high for mutation, to avoid local optima. Tree depth, specifying the maximum depth of GP individuals, ranged from 3 to 5, balancing complexity and computational efficiency. Lastly, we used tournament selection [59], which is currently one of the most widely used methods for selection [60], with a tournament size of 4.

The genetic programming hyperparameters were determined empirically across several experiments, following standard practice in tree-based genetic programming and symbolic regression, with the goal of stable convergence and controlled expression growth. These values regulate the evolutionary search dynamics, including exploration intensity, selection pressure, and bloat control, and do not modify the underlying inverse problem, which is defined by the forward model and the physics-guided fitness functions. Consequently, reasonable variations in these hyperparameters mainly affect convergence rate, computational cost, and the complexity of the evolved expressions, rather than the physical objective being optimized [61,62].

#### 2.3.3. Evaluation of the GP Individuals

**Figure 3:**
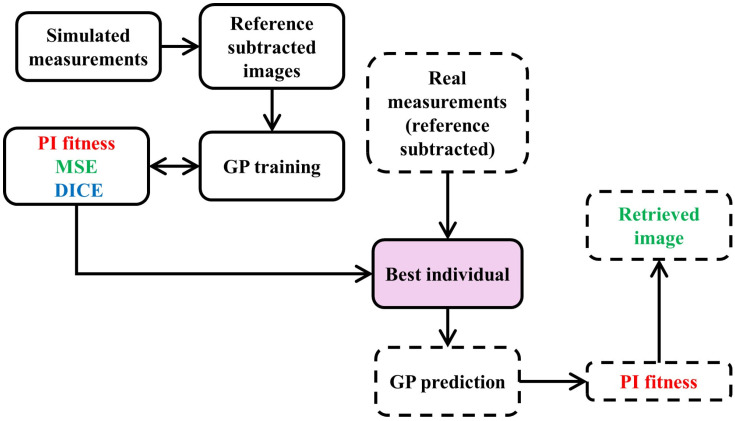
Schematic of the DI-GP workflow. The PI fitness (physics-informed fitness) is used in conjunction with MSE and DICE fitness functions in the training process, where DI-GP evolves a function to reconstruct hidden anomalies inside the sample. However, only the PI fitness (see Equation (7)) is used when the algorithm is tested using unseen experimental data.

The evaluation of GP individuals begins with reference-subtracted simulated measurements, serving as inputs for the GP optimization process, referred to as evolution or training. During this process, explained in the previous subsections, the model undergoes optimization through selection, where various candidate solutions compete over multiple generations, and the best-performing individuals are selected for further refinement. This iterative process enhances the model’s accuracy and robustness. Once the best individual is identified, it is tested on real measurement data to validate its performance. This real-world evaluation ensures that the model’s predictions are accurate and reliable. One important thing to note is that the algorithm leverages ground truth data solely during the training phase. This data is instrumental in optimizing the solution by assessing predictions using the Mean Squared Error (MSE) loss [26], which aids in identifying the distribution of hidden anomalies, and the Dice loss, which enhances edge detection capabilities [63]. The schematic of the DI-GP workflow to evaluate the best individual is shown in Figure 3. All simulations and genetic programming experiments were executed on a workstation-class laptop, namely an HP OMEN by HP Laptop 16 c0004nj, running Microsoft Windows 11 64-bit, equipped with an AMD Ryzen 7 5800H central processing unit with 8 cores and 16 logical processors at a base frequency of 3.2 GHz, and 32 GB of random-access memory. Under this configuration, the full genetic programming evolution required approximately 15 min of wall clock time, and inference for a single reconstruction required less than 1 s.

As can be seen in Figure 3, the fitness function plays a significant role in determining the best individual. It quantifies the accuracy and effectiveness of an individual model in solving the problem at hand by incorporating various performance metrics to provide a comprehensive assessment of the model’s quality. By assigning a fitness score to each candidate, the fitness function guides the selection process, ensuring that the most promising solutions are identified and iteratively refined, ultimately leading to the optimal solution. The fitness functions used in DI-GP are described in the next section.

#### 2.3.4. Fitness Function Used by DI-GP

The cost of the fitness function used by DI-GP to retrieve hidden anomalies in diffuse media is given by:

(6) $$F=\left\{\begin{array}{c} \alpha \xi_{PI}+\beta \xi_{mse}+\gamma \xi_{dice}\;\;\;(training)\\ \xi_{PI}\;\;\;(testing) \end{array}\right.$$

Here, F is the overall cost of evaluating an optimal solution, $\xi_{PI}$ is the cost of the fitness function that is used to optimize Equation (5) and is given by:

(7) $${\xi_{PI}=\left\|M_{predction}-M_{measurement}\right\|}^{2}$$

where $M_{predction}$ is the measurement obtained by the DI-GP prediction and $M_{measurement}$ is the measurement obtained at the camera. By constantly updating $M_{predction}$ using the latest $\chi_{prediction}$ values predicted by the GP function, we incorporate domain knowledge regarding diffuse optical imaging into the algorithm. $\xi_{mse}$ is the MSE loss given by:

(8) $$\xi_{mse}=\left(\frac{1}{N}\right)\sum_{i=1}^{N}\left(\chi_{i}-G_{i}\right)$$

where N is the total number of samples, and $\chi_{i}$ and $G_{i}$ are predictions and the labels, respectively. Similarly, $\xi_{dice}$ is the Dice loss given by:

(9) $$\xi_{dice}=\frac{2\times \sum_{i}^{N}\chi_{i}\times G_{i}}{\sum_{i}^{N}\chi_{i}^{2}+\sum_{i}^{N}G_{i}^{2}}$$

**Figure 4:**
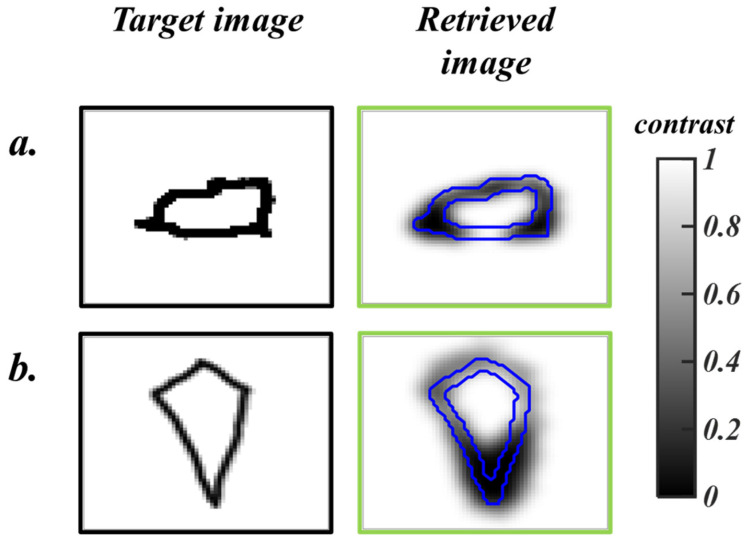
Simulation Results: Image reconstruction using DI-GP on unseen simulated DOI data. The black boxes on the left are the ground truths, and the green boxes show the reconstructions. The color bar depicts the contrast values of the images. (**a**,**b**) show reconstructions for two different samples. The blue borders in the retrieved images show the edges of the ground truth.

α, β, and γ are constant weights used to vary the influence of each fitness function. For the training process, the optimal values for α, β, and γ in this experiment are 0.7, 0.25, and 0.15, respectively. However, when the algorithm is tested using unseen experimental data, β, and γ are 0, and α becomes 1, reducing Equation (6) to $F=\xi_{PI}$. When subjected to unseen simulated data, the DI-GP algorithm yields reconstructions with a mean squared error of 0.029 ± 0.0142. The reconstructed images are shown in Figure 4.

The next sections detail the experimental setup used to validate the DI-GP algorithm and discuss the results.

### 2.4. Experimental Setup to Validate DI-GP

**Figure 5:**
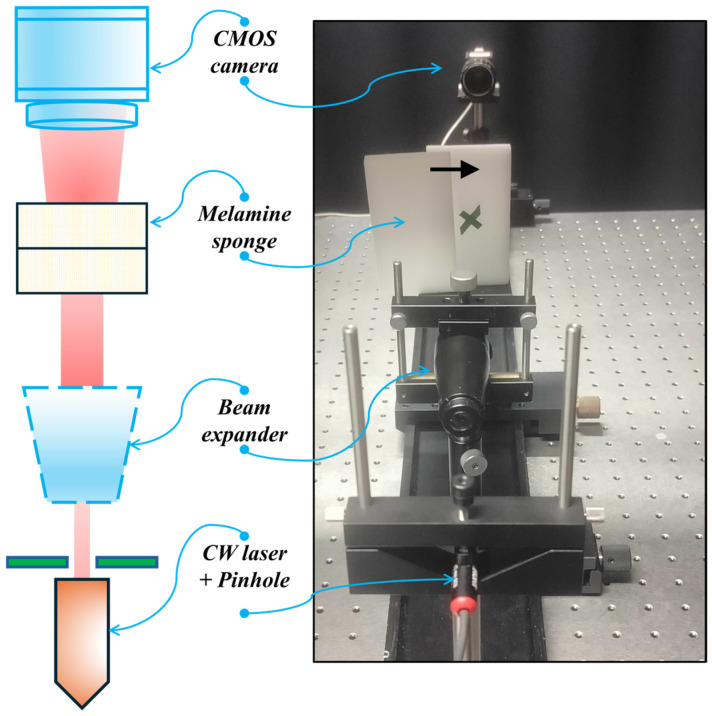
Experimental setup used to detect anomalies hidden inside diffuse media.

This study aims to computationally reconstruct anomalies embedded within the diffuse media using continuous wave (CW) measurements. To achieve this, we build an experimental setup, as illustrated in Figure 5. We use a CW laser source (THORLABS PL252) with a wavelength of 639 nm and an output power of 4.5 mW. The beam is expanded to a spot with a radius of 15 mm using a beam expander, after which it is incident on the melamine foam of 3 cm thickness, containing the anomaly at a depth of 1.5 cm.

As discussed in Section 2.2, the melamine foam is a highly diffuse medium where the measured optical properties correspond to a transport mean free path of 1230 μm, which is more than one order of magnitude smaller than the sample thickness (τ=~24) transport mean free paths. The hidden objects are placed at the central depth of the sample and are random shapes created using black tape.

The light passes through the material, with a portion absorbed by the concealed anomaly. Subsequently, the transmitted light is captured by a CMOS camera (uEYE UI-2210-C), which has a resolution of 640 × 480 pixels and a pixel size of 10 µm. Throughout the image acquisition process, we selectively utilize a resolution of 300 × 300 pixels, enabling the collection of transmitted light from a single source. A noteworthy aspect of these measurement images is the inability to visually discern the presence or absence of an object embedded inside the medium.

The acquired measurements are subsequently resized to 64 × 64 pixels and fed into the DI-GP algorithm, which reconstructs the location and distribution of the hidden object with precision and efficiency. To gauge the efficacy, the performance of our proposed algorithm is compared to that of the conjugate gradient descent algorithm (CGD), a well-established method for image reconstruction and widely used for comparison [13,27,28,29]. The results are detailed in the next section.

## 3. Results and Discussion

**Figure 6:**
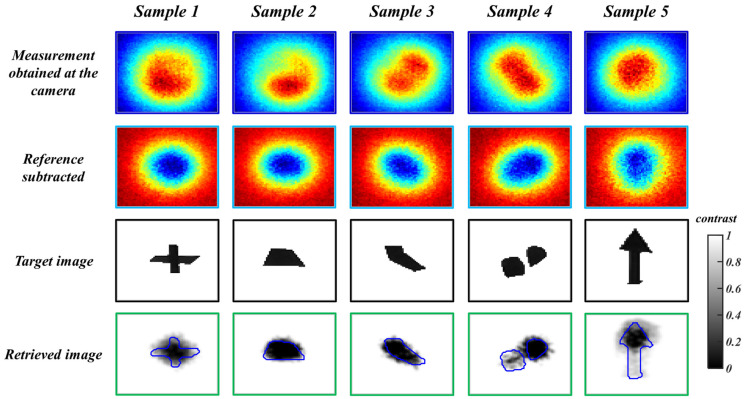
Experimental results: Image reconstruction results from the D-GP algorithm. The first row (in dark blue boxes) shows the recorded images at the CMOS camera. The second row (in light blue boxes) shows the reference-subtracted data. The third row (in black boxes) shows the hidden anomalies inside the diffuse media. The fourth row (in green boxes) shows the retrieved object images obtained using the DI-GP algorithm. The blue borders in the retrieved images show the edges of the ground truth.

Figure 6 showcases the reconstructions obtained through the DI-GP algorithm when tested with experimental measurements. By incorporating knowledge specific to diffuse optical imaging into the fitness evaluation process, the DI-GP algorithm benefits from insights derived from the underlying principles of this field. This comprehensive evaluation approach enhances the robustness and reliability of the evolved solutions, as they are evaluated based on both empirical performance metrics and adherence to known principles of diffuse optical imaging. As depicted in Figure 6, the experimental sample contains a variety of hidden anomalies, including a plus sign, two circles, an arrow, a diagonal line, and a trapezoid. The DI GP algorithm recovers the dominant location and contrast patterns of the concealed objects without prior information regarding their shapes. Fine features and disconnected components can be attenuated when their measurement signatures approach the effective resolution and noise limits of the experimental configuration. This effect is visible in Sample 4, where one of the two circles exhibits reduced recovery, and in Sample 5, where the thin arrow shaft is suppressed while the higher contrast arrow head is retained. Notably, the inference time for this task is less than 1 s.

The effectiveness of the proposed DI-GP algorithm lies in its optimization process, which minimizes the objective function described in Equation (5). Furthermore, by leveraging the fitness function detailed in Equation (6), with parameters α and β set to 0 for experimental evaluation, DI-GP achieves optimal solutions for reconstructing hidden anomalies within the diffuse media. This approach allows the algorithm to adaptively evolve and refine its predictions, leading to accurate reconstructions with high fidelity.

Notably, DI-GP requires only a small dataset for training as it incorporates domain-specific knowledge about DOI, significantly reducing the computational memory and training time needed. This efficient utilization of data resources streamlines the training process. It enhances the algorithm’s scalability and applicability to diverse imaging scenarios.

**Figure 7:**
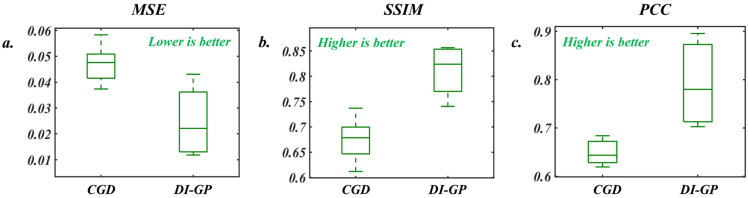
Boxplots comparing the performance of the proposed GI-DP algorithm to that of the analytical method. (**a**–**c**) show the performance of both algorithms in terms of MSE, SSIM, and PCC, respectively.

The performance evaluation of the proposed DI-GP algorithm involves using several key performance metrics to assess the quality and accuracy of the reconstructed images [13,27]. These metrics include Mean Squared Error (MSE), Structural Similarity Index (SSIM), and Pearson Correlation Coefficient (PCC). MSE measures the average squared difference between the pixel values of the reconstructed image and the ground truth image. SSIM quantifies the similarity between two images by comparing the luminance, contrast, and structure. It ranges from −1 to 1, where 1 indicates perfect similarity. Finally, the PCC measures the linear correlation between the pixel values of the reconstructed and ground truth images. The performance achieved by the DI-GP algorithm is 0.0240 ± 0.0121 in terms of MSE, 0.8108 ± 0.0498 in terms of SSIM, and 0.7919 ± 0.0866 in terms of PCC. The low MSE value indicates minimal average reconstruction error, suggesting that the DI-GP algorithm accurately captures the details of the hidden anomalies. The high SSIM and PCC values also reflect strong similarity in contrast, structure, and directional correlation between the reconstructed and ground truth images, affirming the algorithm’s effectiveness in reproducing the true anomalies. The performance of the DI-GP algorithm is compared to that of an Analytical (CGD), and the results are detailed in Figure 7.

The performance enhancement obtained by DI-GP with respect to the analytical solution is given by an improvement ratio defined as:

(10) $$\Lambda =\frac{{met}_{DI-GP}}{{met}_{CGD}}$$

Here, Λ is the improvement ratio. ${met}_{DI-GP}$ and ${met}_{CGD}$ are the performance evaluation values obtained using the performance metrics mentioned above for the DI-GP and the CGD approaches, respectively.

**Figure 8:**
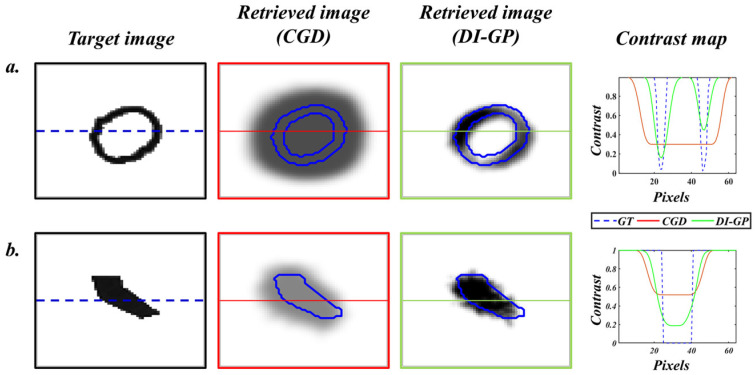
Comparison of image reconstructions using the proposed approach (green boxes) and the analytical approach (red boxes). The blue borders in the retrieved images show the edges of the ground truth. The contrast map on the right compares the pixel intensities in the reconstructions to the ground truth (along the horizontal lines shown in the reconstructions). (**a**) and (**b**) represent examples of two reconstructed images.

The significant improvement ratios obtained for MSE, SSIM, and PCC metrics, namely 0.5138, 1.2015, and 1.2198, respectively, underscore the superior performance of the DI-GP algorithm compared to the analytical solution. These ratios indicate that DI-GP achieves notably lower errors and higher structural similarity and pixel-wise correlation with ground truth images, reflecting its ability to capture better the true characteristics of the hidden anomalies within the diffuse media. Figure 8 compares the reconstructions of the DI-GP and the analytical solution.

While the performance metrics demonstrate that DI GP outperforms conventional analytical reconstructions, it is important to emphasize that deep learning has also delivered impressive advances in diffuse optical imaging when large and diverse training datasets are available. The present work is positioned to complement, rather than compete with, those data-rich approaches by addressing a regime that is frequently encountered in practice, namely, limited training data. Consequently, a quantitative deep learning comparison is not included because the available dataset comprises only 30 samples, which is not sufficient to train, validate, and tune deep neural networks in a manner that would be both reliable and fair. Instead, the manuscript demonstrates that high reconstruction quality can be achieved in small-data settings using a physics-guided genetic programming framework that yields an explicit and interpretable expression. This transparency enables direct examination of the learned reconstruction logic. It supports confidence in deployment, while maintaining competitive performance against established analytical methods in scenarios where data are scarce, and interpretability is a priority.

**Figure 9:**
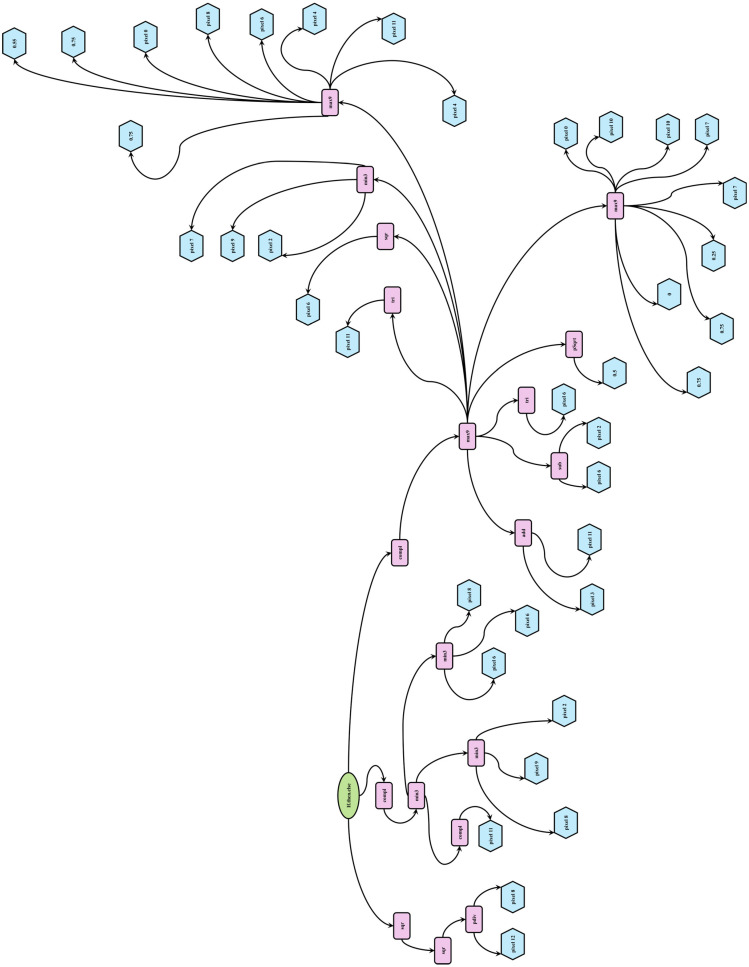
An example of a GP individual evolved by the DI-GP algorithm.

Figure 9 presents a specific GP individual evolved by the DI-GP algorithm, depicted as a tree-structured expression that transforms DOI measurements into clear reconstructions of hidden objects. This evolved expression can be interpreted as a nonlinear contrast-enhancing filter emphasizing sharp transitions and suppressing background noise. It operates by examining local pixel neighborhoods and applying a sequence of adaptive transformations, allowing it to isolate regions likely to correspond to embedded objects within the diffuse media while disregarding more homogeneous background areas.

The GP individual begins with reference-subtracted measurements as inputs, labeled “Pixel #,” corresponding to intensities at different positions. Numeric constants like 0.5 or 0.75 serve as thresholds or scaling factors. These form the tree’s leaf nodes, supplying the raw data needed to generate the reconstruction. Operators, shown as pink rectangles in Figure 9, perform key transformations. Initially, functions like “min3” compute minimum values across several groups of pixels to identify the darkest regions within a local patch. These are combined with inverted signals from key pixels via the “Compl” (Complement) operator to form a dynamic threshold. This result is then inverted again to amplify areas that stand out from their surroundings. Operators such as “sqr” (Square) emphasize stronger signals and suppress weaker ones, “pDiv” (Protected Division) normalizes while avoiding divide-by-zero errors, and “max9” amplifies key variations. Additional arithmetic functions mix, scale, or reshape pixel values.

At the root of the tree, the “if.then.else” node manages adaptive branching, applying different operations based on internal conditions. This design introduces dynamic gating that fine-tunes the reconstruction depending on signal levels. Together, these operators define a nonlinear mapping from measurements to reconstructed pixels, enabling the function to scan and integrate signals across the image. This process untangles scattering effects, converting noisy input into a clearer, high-contrast output.

Rather than acting as a generic filter, the evolved expression encodes a specific heuristic that adaptively responds to spatial intensity patterns typical of hidden anomalies. It behaves similarly to a learned segmentation operator, identifying and boosting subtle signal variations based on their spatial configuration. This level of detailed, functional interpretability reveals how each operator contributes to reconstruction, offering a clear explanation of how predictions are computed, often missing in black-box models. While the evolved expressions can be complex, and further validation on heterogeneous samples is needed, this approach paves the way for DI-GP’s use in clinical imaging. Overall, our study sets an optimistic foundation for the future development of GP in computational diffuse optical imaging.

However, several constraints of the present study limit the current scope and motivate the next research steps. The demonstrated results focus on two-dimensional reconstructions under a fixed acquisition geometry and within the range of optical properties represented in the simulations and phantom experiments. The modelling framework relies on a diffusion-based description of photon transport, which is well suited to highly scattering media but can lose accuracy when diffusion assumptions are weakened, including in strongly heterogeneous tissues. Reconstruction fidelity can also be affected by forward-model mismatch arising from variability in optical parameters, source and detector alignment, and measurement noise statistics relative to those used during training. These considerations motivate future work that expands training and validation across broader distributions of optical properties, geometries, and noise conditions, and that evaluates performance in heterogeneous and anatomically realistic phantoms. Robustness to mismatch can be strengthened through physics-guided fitness designs that incorporate explicit noise models and through data generation protocols that intentionally span experimentally relevant variability. Extension to three-dimensional volumetric imaging is followed by formulating the forward problem on a three-dimensional domain using diffusion-based or Monte Carlo-based light transport, yielding volumetric sensitivities, namely Jacobian operators, that map voxel-wise absorption perturbations to measurement changes. In this regime, genetic programming can be trained to estimate voxel-wise contrast using local volumetric neighborhoods, together with measurement features acquired under multiple illuminations, multiple source-detector separations, or multiple views. The evolutionary procedure remains unchanged, whereas feature construction, target representation, and physics-guided fitness are defined on a volumetric reconstruction grid. The dominant practical challenge is the computational scaling of volumetric forward evaluations and sensitivity operations, which motivates parallel implementations and efficient reduced-order representations.

## 4. Conclusions

In conclusion, the development of DI-GP introduces a novel approach to computational diffuse optical imaging, presenting a potential solution for detecting 2D objects within strongly diffuse media. Leveraging genetic programming and incorporating domain-specific knowledge about DOI, DI-GP offers a method for achieving interpretability algorithms and accurate reconstructions with reduced dataset requirements and training times. Its capability to reconstruct anomalies from real experimental measurements without prior knowledge of their characteristics suggests promising implications for DOI image reconstruction.

Despite some limitations, DI-GP exhibits favorable performance across error metrics compared to analytical algorithms like CGD. Its low error rates in terms of MSE, SSIM, and PCC underscore its efficacy in accurately retrieving information about embedded anomalies. However, translating DI-GP into clinical practice remains a future endeavor, as current experiments have been limited to homogeneous media. Future research should focus on validating DI-GP on inhomogeneous volumetric samples and exploring the integration of neural operators within the GP framework. Such an approach could harness the superior feature extraction capabilities of deep learning while preserving the inherent interpretability of GP, thereby paving the way for more robust and clinically applicable imaging solutions.

## Figures and Tables

**Table 1:** Pseudocode showing the generation of a predicted image.

1. function GenerateOutputImage(M, GP_individual, N) -> X_prediction: 2. X_prediction = createImageWithSameSizeAs(M) 3. 4. for each row in M: 5. for each column in M: 6. environment = extractEnvironment(M, row, column, N) 7. if isValidEnvironment(environment): 8. outputPixel = applyGPIndividual(environment, GP_individual) 9. setPixel(X_prediction, row, column, outputPixel) 10. else: 11. continue 12. return X_prediction

**Table 2:** List of inner functions and terminals utilized in the GP algorithm.

Category	Inner-Functions
Arithmetic-Binary	$Add\left(x,y\right),$ $Subtract\left(x,y\right),$ $Multiply\left(x,y\right),$ ProtectedDivision(x,y)
Arithmetic-Unary	$Minus\left(x\right)=-x,$ $Square(x)=x^{2},$ $Tri(x)=x^{3},$ SquareRoot(x)=sign(x)×√abs(x), Complement(x)=1−x
Minimum and Maximum	$Min3\left(x1,x2,\ldots ,x9\right),$ $Min6\left(x1,x2,\ldots ,x9\right),$ $Min9\left(x1,x2,\ldots ,x9\right),$ $Max3\left(x1,x2,\ldots ,x9\right),$ $Max6\left(x1,x2,\ldots ,x9\right),$ $Max9\left(x1,x2,\ldots ,x9\right)$
Conditional	If_Then_Else(x,y,z) = y if x>0.5 else z If_Larger(x,y,z,t) = z if x>y else t If_In_Range(x,y,z,t,w)=t if x≤y<z else w
**Category**	**Terminals**
Pixels	Pixel1, …, ${Pixel}_{N\times N}$
Constants	0.0,0.25,0.5,0.75,1.0

## Data Availability

Data underlying the results presented in this paper are not publicly available at this time. However, they may be obtained from the corresponding author (GMB) upon reasonable request.
